# On a New Three-Step Class of Methods and Its Acceleration for Nonlinear Equations

**DOI:** 10.1155/2014/134673

**Published:** 2014-09-03

**Authors:** T. Lotfi, K. Mahdiani, Z. Noori, F. Khaksar Haghani, S. Shateyi

**Affiliations:** ^1^Department of Mathematics, Islamic Azad University, Hamedan Branch, Hamedan, Iran; ^2^Department of Mathematics, Islamic Azad University, Shahrekord Branch, Shahrekord, Iran; ^3^Department of Mathematics and Applied Mathematics, School of Mathematical and Natural Sciences, University of Venda, Thohoyandou 0950, South Africa

## Abstract

A class of derivative-free methods without memory for approximating a simple zero of a nonlinear equation is presented. The proposed class uses four function evaluations per iteration with convergence order eight. Therefore, it is an optimal three-step scheme without memory based on Kung-Traub conjecture. Moreover, the proposed class has an accelerator parameter with the property that it can increase the convergence rate from eight to twelve without any new functional evaluations. Thus, we construct a with memory method that increases considerably efficiency index from 8^1/4^ ≈ 1.681 to 12^1/4^ ≈ 1.861. Illustrations are also included to support the underlying theory.

## 1. Introduction

The first attempts for classifying iterative root-finding methods were done by Traub [1]. He divided iterative methods for finding zeros of a function into two sets: one-point and multipoint methods. There is a fact about how to create a new method. As Traub investigated in his book [1], and Kung and Traub mentioned in [2], construction of one-point methods is not a useful task. In other words, to construct an optimal one-point method with convergence order *n*, we need *n* functional evaluations, while for construction an optimal method without memory having convergence order 2^*n*^; only *n* + 1 function evaluations are required.

To be more precise, constructing an optimal one-point method with eighth-order convergence needs eight function evaluations, while constructing an optimal three-point method without memory having the same convergence order requires four functional evaluations. As a result, many researchers have paid much attention to construct optimal multipoint iterations without memory based on the unproved conjecture due to Kung and Traub: any multipoint iteration without memory using *n* + 1 function evaluations can reach the optimal order 2^*n*^.

This work follows two main goals: frst developing a new optimal three-step derivative-free class of methods without memory and second developing the proposed class to methods with memory. In this way, it reaches convergence order 12 without any new functional evaluations. Because of the derivative-free property of the proposed class, it can be used for finding zeros of not only smooth functions but also nonsmooth ones. Moreover, as we pointed out above we can reach the convergence order 12 using the same functional evaluations (to three-step without memory iterations) and, therefore, increasing 50% convergence order is the other aspect and contribution of this work.

Note that in most test problems for nonlinear equations computing derivatives are an easy exercise. However, for some practical problems computing the derivative might be a cumbersome task and we have to relay on methods free of derivatives. For further reading on this topic, one may refer to [3–6].

The paper is organized as follows. First, a new without memory family of optimal order eight, consuming four function evaluations per iteration, is proposed by using two weight functions in Section 2. A different way to compute the order of convergence for iterative methods that use divided differences instead of derivatives is presented in Section 3, when we derive a method with memory. The significant increase of convergence speed is achieved without additional function evaluations, which is the main advantage of such methods.  Section 4 is devoted to numerical results connected to the order of the methods with and without memory. And finally, concluding remarks will be drawn in Section 5.

## 2. Construction of a New Three-Step Class

Let the scalar function *f* : *D* ⊂ *R* → *R* and *f*(*α*) = 0 ≠ *f*′(*α*) = *c*_1_. In other words, *α* is a simple zero of *f*(*x*) = 0. In this section, we start with the three-step scheme:

(1) $$\begin{array}{c} y_{k}=x_{k}-\frac{f\left(x_{k}\right)}{f^{\prime }\left(x_{k}\right)},\\ z_{k}=y_{k}-\frac{f\left(y_{k}\right)}{f^{\prime }\left(y_{k}\right)},\\ x_{k+1}=z_{k}-\frac{f\left(z_{k}\right)}{f^{\prime }\left(z_{k}\right)}, \end{array}$$

where *k* = 0,1,…, is the iteration indicator. The order of convergence for (1) is eight but its computational efficiency is low. We substitute derivatives in all three steps by suitable approximations that use available data; thus we introduce the following approximations:

(2) f′(xk)≈f[xk,wk],where  wk=xk+βf(xk),f′(yk)≈f[yk,wk]H(uk,vk), uk=f(yk)f(xk),  vk=f(yk)f(wk),f′(zk)≈f[yk,zk]+f[wk,yk,zk](zk−yk)W(sk),sk=f(zk)f(xk),

in the first, second, and third steps of (1), where *H* and *W* are weight functions. The following iterative family of three-step methods is obtained:

(3) yk=xk−f(xk)f[xk,wk], wk=xk+βf(xk),β∈R,zk=yk−H(uk,vk)f(yk)f[yk,wk], uk=f(yk)f(xk),vk=f(yk)f(wk),xk+1=zk−W(sk)×f(zk)f[zk,yk]+f[wk,yk,zk](zk−yk),sk=f(zk)f(xk).

In the following theorem, we state suitable conditions for deriving a new optimal class without memory according to the Kung and Traub conjecture [2] (also known as K-T hypothesis).

**Theorem 1 .**

Let *f* : *D* ⊂ *R* → *R* be a scalar function which has a simple root *α* in the open interval *D*, and also the initial approximation *x*_0_ is sufficiently close to the simple zero. Then, the three-step iterative method (3) has eighth-order under the conditions *W*(0) = *W*′(0) = 1, *H*(0,0) = *H*_*u*_(0,0) = *H*_*uu*_(0,0) = 1, *H*_*v*_(0,0) = *H*_*vv*_(0,0) = 0, and *H*_*uv*_(0,0) = 2 and satisfies the following error equation:

(4) ek+1=(1+βf′(α))4c22c3 ×((9+βf′(α)(7+βf′(α)))c23+2c2c3−c4)ek8+O(ek9).

**Proof**

By using Taylor's expansion of *f*(*x*) around *α* and taking into account that *f*(*α*) = 0, we obtain

(5) f(x)=f′(α)(ek+c2ek2+c3ek3+c4ek4+c5ek5+c6ek6+c7ek7+c8ek8+O(ek9)),

where *e*_*k*_ = *x*_*k*_ − *α*, *e*_*k*,*w*_ = *w*_*k*_ − *α*, *e*_*k*,*y*_ = *y*_*k*_ − *α*, *e*_*k*,*z*_ = *z*_*k*_ − *α*, and *c*_*k*_ = *f* ^(*k*)^(*α*)/*k*!*f*′(*α*). Therefore,

(6) ek,w=(1+βf′(α))ek+βf′(α)c2ek2+βf′(α)c3ek3+βf′(α)c4ek4+βf′(α)c5ek5+βf′(α)c6ek6+βf′(α)c7ek7+βf′(α)c8ek8+O(ek9),

(7) f(wk)=f′(α)(1+f′(α)β)ek+f′(α)(1+f′(α)β(3+f′(α)β))c2ek2+⋯+O(ek9).

Note that we used ⋯ in order to avoid writing further terms of the Taylor expansions due to symbolic computations. By using (5) and (7), we obtain

(8) f[xk,wk]=f′(α)(1+(2+βf′(α)))c2ek  +f′(α)(βf′(α)c22+(3+βf′(α)      ×(3+βf′(α)))c3)ek2 +⋯+O(ek8).

Dividing (5) by (8) gives us

(9) f(xk)f[xk,wk]=ek−(1+βf′(α))c2ek2 +((2+βf′(α)(2+βf′(α)))c22  −(1+βf′(α))(2+βf′(α))c3)ek3 +⋯+O(ek9).

And thus,

(10) $$\begin{array}{c} e_{k,y}=-\;{\left(1+\beta f^{\prime }\left(\alpha \right)\right)}^{2}c_{2}c_{3}e_{k}^{4}+\cdots +O\left(e_{k}^{9}\right). \end{array}$$

Subsequently, we have

(11) f[yk,wk]=f′(α)+f′(α)(1+βf′(α))c2ek+f′(α)((1+2βf′(α))c22+(1+βf′(α))2c3)ek2+⋯+O(ek8).

Thus,

(12) h(uk,vk)f(yk)f[yk,wk]=(1+βf′(α))c2ek2+(−(2+βf′(α)(2+βf′(α)))c22 +(1+βf′(α))(2+βf′(α))c3)×ek3+⋯+O(ek9),f[wk,yk,zk]=f′(α)c2+f′(α)(1+βf′(α))c3ek +f′(α)((1+2βf′(α))c2c3      +(1+βf′(α))2c4)ek2 +⋯+O(ek9).

Therefore, we attain

(13) f(zk)f[zk,yk]+f[wk,yk,zk](zk−yk)  =− (1+βf′(α))2c2c3ek4+⋯+O(ek9).

Finally, according to the above analysis, the general error equation is given by

(14) ek+1=ek,z−W(sk)f(zk)f[zk,yk]+f[wk,yk,zk](zk−yk)=(1+βf′(α))4c22c3 ×((9+βf′(α)(7+βf′(α)))c23  +2c2c3−c4)ek8+O(ek9),

so that the proof of the theorem is finished.

We provide some specific weight functions that satisfy the conditions of Theorem 1 as follows:

(15) $$\begin{array}{c} H_{1}\left(u_{k},v_{k}\right)=1+u_{k}+2u_{k}v_{k}+u_{k}^{2},\\ H_{2}\left(u_{k},v_{k}\right)=\frac{1}{1-u_{k}-2u_{k}v_{k}},\\ W_{1}\left(s_{k}\right)=\cos\left(s_{k}\right)+\sin\left(s_{k}\right),\\ W_{2}\left(s_{k}\right)=\frac{1}{1-s_{k}},\\ W_{3}\left(s_{k}\right)=1+s_{k},\\ W_{4}\left(s_{k}\right)=e^{s_{k}}. \end{array}$$

We consider these weight functions in, without and with memory methods, (3) and (18) in the forthcoming sections.

There are some measures for comparing various iterative techniques. Traub [1] introduced the informational efficiency and efficiency index, which can be expressed in terms of the order (*r*) of the method and the number of function (and derivative) evaluations (*ρ*). In fact, the efficiency index (or computational efficiency) is given by *E* = *r* ^1/*ρ*^.

Clearly, the efficiency index of the proposed optimal class of method is 8^1/4^ ≈ 1.682 which is optimal in the sense of K-T hypothesis and is higher than two- or one-step methods without memory.

It is worth emphasizing that the maximal order of convergence is not the only goal in constructing root-finding methods and, consequently, the ultimate measure of efficiency of the designed method. Complexity of the formulae involved, often called combinatorial cost, makes another important parameter, which should be taken into account. Hence, we wish to construct a new method with memory possessing a high order 12 requiring only 4 functional evaluations (just like (3)).

In the next section, we will modify the proposed method and introduce a new method. We use an accelerator parameter to increase the order of convergence significantly.

## 3. Construction of a Method with Memory

Error equation (14) indicates that the order of convergence for class (3) is equal to eight. This section is concerned with extracting an efficiency with memory method from (3) since its error equation contains the parameter *β* which can be approximated in such a way that increases the local order of convergence.

We set *β* = *β*_*k*_ as the iteration proceeds by the formula $\beta_{k}=-1/\bar{f^{\prime }}(\alpha )$ for *k* = 1,2,…, where $\bar{f^{\prime }}(\alpha )$ is an approximation of *f*′(*α*). We have a method via the following forms of *β*_*k*_:

(16) $$\begin{array}{c} \beta_{k}=-\frac{1}{\bar{f^{\prime }}\left(\alpha \right)}=-\frac{1}{N_{4}^{\prime }\left(x_{k}\right)}. \end{array}$$

The key idea that provides the order acceleration lies in a special form of the error relation and a convenient choice of a free parameter. We define a self-accelerating parameter, which is calculated during the iterative process using Newton's interpolating polynomial.

Hence, we consider Newton's interpolation as the method for approximating *f*′(*α*), where *N*_4_(*t*) is Newton's interpolation polynomial of fourth degree, set through five available approximations *x*_*k*_, *z*_*k*−1_, *y*_*k*−1_, *w*_*k*−1_, *x*_*k*−1_ as follows:

(17) N4′(xk)=[ddtN4(t)]t=xk=[ddt(f(xk)+f[xk,zk−1](t−xk) +f[xk,zk−1,yk−1](t−xk) ×(t−zk−1)+f[xk,zk−1,yk−1,xk−1](t−xk) ×(t−zk−1)(t−yk−1) +f[xk,zk−1,yk−1,xk−1,wk−1] ×(t−xk)(t−zk−1)(t−yk−1) ×(t−xk−1))]t=xk=f[xk,zk−1]+f[xk,zk−1,yk−1](xk−zk−1) +f[xk,zk−1,yk−1,xk−1](xk−zk−1)(xk−yk−1) +f[xk,zk−1,yk−1,xk−1,wk−1](xk−zk−1) ×(xk−yk−1)(xk−xk−1).

Here, the with memory development of (3) can be presented as follows:

(18) yk=xk−f(xk)f[xk,wk], wk=xk+βkf(xk),zk=yk−H(uk,vk)f(yk)f[yk,wk], uk=f(yk)f(xk),vk=f(yk)f(wk),xk+1=zk−W(sk)×f(zk)f[zk,yk]+f[wk,yk,zk](zk−yk)sk=f(zk)f(xk).

We attempt to prove that the method with memory (18) has convergence order twelve provided that we use accelerator *β*_*k*_ as in (16). It should be remarked that we have applied the Herzberger's matrix method [7].

**Theorem 2 .**

If an initial approximation *x*_0_ is sufficiently close to the zero *α* of *f*(*x*) and the parameter *β*_*k*_ in the iterative scheme (18) is recursively calculated by the forms given in (16), then the order of convergence is twelve.

**Proof**

We will use Herzberger's matrix method to determine the order of convergence. Note that the lower bound of order for a single-step *s*-point method *x*_*k*_ = *G*(*x*_*k*−1_, *x*_*k*−2_,…, *x*_*k*−*s*_) is the spectral radius of a matrix *M* ^(*s*)^ = (*m*_*ij*_), associated to the method with elements:

(19) m1,j=amount  of  information  required  at  point  xk−jj=1,2,…,s,mi,i−1=1 (i=2,3,…,s),mi,j=0, otherwise.

On the other hand, the lower bound of order of an *s*-step method *G* = *G*_1_∘*G*_2_∘⋯∘*G*_*s*_ is the spectral radius of the product of matrices *M* = *M*_1_ · *M*_2_ ⋯ *M*_*s*_.

We can express each approximation *x*_*k*+1_, *z*_*k*_, *y*_*k*_, and *w*_*k*_ as a function of available information *f*(*z*_*k*_), *f*(*y*_*k*_), *f*(*w*_*k*_), and *f*(*x*_*k*_) from the *k*th iteration and *f*(*z*_*k*−1_), *f*(*y*_*k*−1_), *f*(*w*_*k*−1_), and *f*(*x*_*k*−1_) from the previous iteration, depending on the accelerating technique. Now, we determine the order of convergence for (18) applied for the calculation of *β*_*k*_.

*Method (N4)*. We use the following matrices to express informational dependence:

(20) $$\begin{array}{c} x_{k+1}=\varphi_{1}\left(z_{k},y_{k},w_{k},x_{k},z_{k-1}\right)⟶M_{1}=\left[\begin{array}{ccccc} 1 & 1 & 1 & 1 & 0\\ 1 & 0 & 0 & 0 & 0\\ 0 & 1 & 0 & 0 & 0\\ 0 & 0 & 1 & 0 & 0\\ 0 & 0 & 0 & 1 & 0 \end{array}\right],\\ z_{k}=\varphi_{2}\left(y_{k},w_{k},x_{k},z_{k-1},y_{k-1}\right)⟶M_{2}=\left[\begin{array}{ccccc} 1 & 1 & 1 & 0 & 0\\ 1 & 0 & 0 & 0 & 0\\ 0 & 1 & 0 & 0 & 0\\ 0 & 0 & 1 & 0 & 0\\ 0 & 0 & 0 & 1 & 0 \end{array}\right],\\ y_{k}=\varphi_{3}\left(w_{k},x_{k},z_{k-1},y_{k-1},w_{k-1}\right)⟶M_{3}=\left[\begin{array}{ccccc} 1 & 1 & 0 & 0 & 0\\ 1 & 0 & 0 & 0 & 0\\ 0 & 1 & 0 & 0 & 0\\ 0 & 0 & 1 & 0 & 0\\ 0 & 0 & 0 & 1 & 0 \end{array}\right],\\ w_{k}=\varphi_{4}\left(x_{k},z_{k-1},y_{k-1},w_{k-1},x_{k-1}\right)⟶M_{4}=\left[\begin{array}{ccccc} 1 & 1 & 1 & 1 & 0\\ 1 & 0 & 0 & 0 & 0\\ 0 & 1 & 0 & 0 & 0\\ 0 & 0 & 1 & 0 & 0\\ 0 & 0 & 0 & 1 & 0 \end{array}\right]. \end{array}$$

Hence, it is easy to derive

(21) $$\begin{array}{c} M^{\left(N4\right)}=M_{1}M_{2}M_{3}M_{4}=\left[\begin{array}{ccccc} 8 & 4 & 4 & 4 & 4\\ 4 & 2 & 2 & 2 & 2\\ 2 & 1 & 1 & 1 & 1\\ 1 & 1 & 1 & 1 & 1\\ 1 & 0 & 0 & 0 & 0 \end{array}\right], \end{array}$$

with the eigenvalues {12,0, 0,0, 0}. Consequently, the order of the method with memory (18)-(*N*4) is at least twelve. The proof of Theorem 2 is finished.

Clearly, the proposed with memory scheme possesses a high computational efficiency index 12^1/4^ ≈ 1.861, which makes it interesting for practical problems.

## 4. Numerical Examples

In this section, we test our proposed methods and compare their results with some other methods of the same order of convergence. The errors |*x*_*k*_ − *α*| denote approximations to the sought zeros, and *a*(−*b*) stands for *a* × 10^−*b*^. Moreover, coc indicates computational order of convergence and is computed by

(22) $$\begin{array}{c} \text{coc}=\frac{\log\left(\left|f\left(x_{k}\right)/f\left(x_{k-1}\right)\right|\right)}{\log\left(\left|f\left(x_{k-1}\right)/f\left(x_{k-2}\right)\right|\right)}. \end{array}$$

The calculated value coc estimates the theoretical order of convergence well when “pathological behavior” of the iterative method (for instance, slow convergence at the beginning of the implemented iterative method, “oscillating” behavior of approximations, etc.) does not exist.

We have used 1000-fixed floating point arithmetic so as to minimize the effect of round-off errors.

By using weight functions (15), we introduce some concrete methods based on the proposed class. Note that it is assumed that the initial estimate *β*_0_ should be chosen before starting the iterative process and also *x*_0_ is given suitably.

Concrete method 1:

(23) yk=xk−f(xk)f[xk,wk], wk=xk+βkf(xk),zk=yk−(1+uk+2ukvk+uk2)f(yk)f[yk,wk],uk=f(yk)f(xk),vk=f(yk)f(wk),xk+1=zk−(cos⁡(sk)+sin(sk))×f(zk)f[zk,yk]+f[wk,yk,zk](zk−yk),sk=f(zk)f(xk).

Concrete method 2:

(24) yk=xk−f(xk)f[xk,wk], wk=xk+βkf(xk),zk=yk−(1+uk+2ukvk+uk2)f(yk)f[yk,wk],uk=f(yk)f(xk),vk=f(yk)f(wk),xk+1=zk−(11−sk)×f(zk)f[zk,yk]+f[wk,yk,zk](zk−yk),sk=f(zk)f(xk).

Concrete method 3:

(25) yk=xk−f(xk)f[xk,wk], wk=xk+βkf(xk),zk=yk−(1+uk+2ukvk+uk2)f(yk)f[yk,wk],uk=f(yk)f(xk),vk=f(yk)f(wk),xk+1=zk−(1+sk) ×f(zk)f[zk,yk]+f[wk,yk,zk](zk−yk),sk=f(zk)f(xk).

Concrete method 4:

(26) yk=xk−f(xk)f[xk,wk], wk=xk+βkf(xk),zk=yk−(1+uk+2ukvk+uk2)f(yk)f[yk,wk],uk=f(yk)f(xk),vk=f(yk)f(wk),xk+1=zk−(esk)f(zk)f[zk,yk]+f[wk,yk,zk](zk−yk),sk=f(zk)f(xk).

Concrete method 5:

(27) yk=xk−f(xk)f[xk,wk], wk=xk+βkf(xk),zk=yk−(11−uk−2ukvk)f(yk)f[yk,wk],uk=f(yk)f(xk),vk=f(yk)f(wk),xk+1=zk−(cos⁡(sk)+sin(sk))×f(zk)f[zk,yk]+f[wk,yk,zk](zk−yk)sk=f(zk)f(xk).

Concrete method 6:

(28) yk=xk−f(xk)f[xk,wk], wk=xk+βkf(xk),zk=yk−(11−uk−2ukvk)f(yk)f[yk,wk],uk=f(yk)f(xk),vk=f(yk)f(wk),xk+1=zk−(11−sk)×f(zk)f[zk,yk]+f[wk,yk,zk](zk−yk),wk=f(zk)f(xk).

Concrete method 7:

(29) yk=xk−f(xk)f[xk,wk], wk=xk+βkf(xk),zk=yk−(11−uk−2ukvk)f(yk)f[yk,wk],uk=f(yk)f(xk),vk=f(yk)f(wk),xk+1=zk−(1+sk)×f(zk)f[zk,yk]+f[wk,yk,zk](zk−yk),wk=f(zk)f(xk).

Concrete method 8:

(30) yk=xk−f(xk)f[xk,wk], wk=xk+βkf(xk),zk=yk−(11−uk−2ukvk)f(yk)f[yk,wk],uk=f(yk)f(xk),vk=f(yk)f(wk),xk+1=zk−(esk)×f(zk)f[zk,yk]+f[wk,yk,zk](zk−yk),wk=f(zk)f(xk).

Several iterative methods (IM) of optimal order eight, which also require four function evaluations, for comparisons with our proposed methods have been chosen.

Three-step method by Wang et al. [8]:

(31) $$\begin{array}{c} y_{k}=x_{k}-\frac{f\left(x_{k}\right)}{f\left[x_{k},w_{k}\right]},\;w_{k}=x_{k}+\beta f\left(x_{k}\right),\\ z_{k}=y_{k}-\frac{f\left(y_{k}\right)}{f\left[y_{k},w_{k}\right]}H\left(t_{k}\right),\;t_{k}=\frac{f\left(y_{k}\right)}{f\left(x_{k}\right)},\\ x_{k+1}=z_{k}-\frac{f\left(z_{k}\right)}{f\left[z_{k},w_{k}\right]}G\left(t_{k},s_{k}\right),\;s_{k}=\frac{f\left(z_{k}\right)}{f\left(y_{k}\right)}, \end{array}$$

where functions *H* and *G* are following *H*_1_(*t*) = 1 + *t*, *G*_1_(*t*, *s*) = 1 + *t* + *s* + 2*ts* − (1 + *λ*)*t* ^3^, and  *λ* = 1/(1 + *βf*[*x*, *w*]).

Three-step method by Lotfi and Tavakoli [9]:

(32) yn=xn−f(xn)f[xn,wn],zn=yn−H(tn,un)f(yn)f[yn,wn],tn=f(y)f(x),un=f(w)f(x),xk+1=zn−G(tn,sn)W(vn,sn)f(zn)f[zn,wn],sn=f(z)f(y),vn=f(z)f(x),

where

(33) W(sn,vn)=1+sn2+vn2,G(tn,sn)=1+tn+sn+2tnsn+(−1−ϕn)tn3,(ϕn=11+βnf[xn,wn]),H(tn,un)=1+tn.

Derivative-free Kung-Traub's family [2]:

(34) yk=xk−f(xk)f[xk,wk], wk=xk+βf(xk),zk=yk−f(yk)f(wk)[f(wk)−f(yk)]f[xk,yk],xk+1=zk−f(yk)f(wk)(yk−xk+f(xk)/f[xk,zk])[f(yk)−f(zk)][f(wk)−f(zk)]+f(yk)f[yk,zk].

Three-step methods made by Zheng et al. [10]:

(35) yk=xk−f(xk)f[xk,wk], wk=xk+βf(xk),zk=yk−f(yk)f[yk,wk]+f[yk,xk,wk](yk−xk),xk+1=zk−(f(zk)×(f[zk,yk]+f[zk,yk,xk](zk−yk)        +f[zk,yk,xk,wk](zk−yk)        ×(zk−xk))−1).

In Tables 1, 3, and 5 our without memory proposed method by different weight functions (23)–(30) has been compared with optimal three-point methods (34) and (35), and we observe that all these methods behave very well practically and confirm their theoretical results.

Also Tables 2, 4, and 6 present numerical results for our with memory classes (23)–(30). It is also clear that all these methods behave very well practically and confirm their relevant theories. They all provide wherea bout twelve of convergence order.

## 5. Concluding Remarks

We have constructed a class of methods without and with memory. Our proposed methods do not need any derivative and therefore are applicable to nonsmooth functions too. Another advantage of the proposed methods is that their without memory versions are optimal in the sense of K-T conjecture. In addition, it contains an accelerator parameter which rises convergence order from eight to twelve without any new functional evaluations. In other words, the efficiency index of the with memory class is 12^1/4^ ≈ 1.861.

We finalize this work by suggesting some outlines for future research: first developing the proposed methods for computing multiple roots and second exploring its dynamic or basins of attractions, and finally we wonder why not to use an adaptive arithmetic in each step of the iterative method instead of using a fixed precision, since this higher precision is only necessary in the last step of the iterative process.

## Figures and Tables

**Table 1:** Consider *f*_1_(*x*) = sin⁡⁡(π*x*)*e* ^(*x*^2^+*x*cos⁡(*x*)−1)^ + *x*log⁡(*x*sin⁡⁡(*x*) + 1), α = 0, *x*_0_ = 0.6, β = −0.01.

Methods without memory	|*x*_1_ − α|	|*x*_2_ − α|	|*x*_3_ − α|	coc
Our method (23)	5.3810(−3)	5.5392(−25)	6.9091(−201)	8.0003
Our method (24)	2.1802(−3)	3.9973(−28)	5.0816(−226)	8.0001
Our method (25)	4.2865(−3)	8.9612(−26)	3.2420(−207)	8.0002
Our method (26)	3.1912(−3)	8.4384(−27)	2.0042(−215)	8.0002
Our method (27)	5.7030(−3)	4.9625(−25)	1.6203(−201)	8.0002
Our method (28)	2.3293(−3)	3.8278(−28)	2.0304(−226)	8.0001
Our method (29)	5.7030(−3)	4.9625(−25)	1.6203(−201)	8.0002
Our method (30)	4.5488(−3)	8.1186(−26)	8.3149(−208)	8.0002
(34), β = −0.01	9.7732(−3)	4.3543(−24)	6.6317(−195)	8.0002
(35), β = −0.01	1.7342(−2)	1.1724(−21)	3.5692(−175)	8.0085

**Table 2:** Consider *f*_1_(*x*) = sin⁡⁡(π*x*)*e* ^(*x*^2^+*x*cos⁡(*x*)−1)^ + *x*log⁡(*x*sin⁡⁡(*x*) + 1), α = 0, *x*_0_ = 0.6, β_0_ = −0.01.

Methods with memory	|*x*_1_ − α|	|*x*_2_ − α|	|*x*_3_ − α|	coc
Our method (23)	5.3810(−3)	2.7788(−35)	1.8668(−426)	12.1156
Our method (24)	2.1802(−3)	3.0336(−38)	5.3469(−462)	12.1571
Our method (25)	4.2865(−3)	5.2041(−36)	3.4739(−435)	12.1273
Our method (26)	3.1912(−3)	5.6445(−37)	9.2065(−447)	12.1411
Our method (27)	5.7030(−3)	4.6138(−35)	8.1591(−424)	12.1138
Our method (28)	2.3293(−3)	5.4580(−38)	6.1228(−459)	12.1556
Our method (29)	4.5488(−3)	8.7828(−36)	1.8467(−432)	12.1256
Our method (30)	3.3939(−3)	9.7470(−37)	6.4438(−444)	12.1395
(31), β = −1	2.1170(−3)	1.1077(−41)	3.4407(−501)	12.0035
(32), β = −1	5.7578(−3)	2.7125(−39)	1.4859(−474)	11.9819

**Table 3:** Consider *f*_2_(*x*) = *e* ^−5*x*^(*x* − 2)(*x* ^10^ + *x* + 2), α = 2, *x*_0_ = 2.2, β = −1.

Methods without memory	|*x*_1_ − α|	|*x*_2_ − α|	|*x*_3_ − α|	coc
Our method (23)	5.4211(−6)	7.6321(−54)	1.1776(−436)	8.0000
Our method (24)	5.4981(−6)	8.5429(−54)	2.9020(−436)	8.0000
Our method (25)	5.4468(−6)	7.9261(−54)	1.5935(−436)	8.0001
Our method (26)	5.4725(−6)	8.2301(−54)	2.1533(−436)	8.0000
Our method (27)	3.2606(−6)	1.3070(−55)	8.7125(−451)	8.0000
Our method (28)	3.3757(−6)	1.7249(−55)	8.0153(−450)	8.0000
Our method (29)	3.2991(−6)	1.4354(−55)	1.8434(−450)	8.0000
Our method (30)	3.3375(−6)	1.5747(−55)	3.8666(−450)	8.0000
(34), β = −1	8.0552(−6)	1.4131(−52)	1.2677(−456)	8.0000
(35), β = −0.01	3.9453(−6)	1.2705(−59)	5.0021(−491)	8.0085

**Table 4:** Consider *f*_2_(*x*) = *e* ^−5*x*^(*x* − 2)(*x* ^10^ + *x* + 2), α = 2, *x*_0_ = 2.2, β_0_ = −1.

Methods with memory	|*x*_1_ − α|	|*x*_2_ − α|	|*x*_3_ − α|	coc
Our method (23)	5.4211(−6)	2.1857(−80)	4.4911(−970)	11.9590
Our method (24)	5.4981(−6)	2.4529(−80)	1.7931(−969)	11.9587
Our method (25)	5.4468(−6)	2.2719(−80)	7.1443(−970)	11.9589
Our method (26)	5.4725(−6)	2.3611(−80)	9.2065(−969)	11.9588
Our method (27)	5.4981(−6)	2.4529(−80)	1.7931(−969)	11.9587
Our method (28)	3.3757(−6)	7.7624(−82)	1.8084(−987)	11.9732
Our method (29)	3.2991(−6)	6.4445(−82)	1.9393(−988)	11.9737
Our method (30)	3.3375(−6)	7.0781(−82)	5.9748(−988)	11.9735
(31), β = −1	4.9425(−5)	1.5275(−69)	4.1256(−874)	12.0535
(32), β = −1	4.0518(−6)	8.2730(−77)	2.4267(−861)	11.0982

**Table 5:** Consider *f*_3_(*x*) = *e* ^*x*^3^−*x*^ − cos⁡(*x* ^2^ − 1) + *x* ^3^ + 1, α = −1, *x*_0_ = −1.65, β = −1.

Methods without memory	|*x*_1_ − α|	|*x*_2_ − α|	|*x*_3_ − α|	coc
Our method (23)	6.0256(−3)	4.0898(−24)	1.8272(−193)	8.0001
Our method (24)	5.9797(−3)	3.8471(−24)	1.1202(−193)	8.0001
Our method (25)	6.0104(−3)	4.0081(−24)	1.5548(−193)	8.0001
Our method (26)	5.9952(−3)	3.9278(−24)	1.3225(−193)	8.0001
Our method (27)	5.3628(−3)	1.6071(−24)	1.0388(−196)	8.0001
Our method (28)	5.3357(−3)	1.5431(−24)	7.5042(−197)	8.0001
Our method (29)	5.3537(−3)	1.5856(−24)	9.3292(−197)	8.0001
Our method (30)	5.3448(−3)	1.5644(−24)	8.3766(−197)	8.0001
(34), β = −1	2.9152(−4)	5.3239(−34)	6.5827(−272)	8.0000
(35), β = −0.01	6.4036(−5)	4.4335(−44)	2.4457(−357)	7.9995

**Table 6:** Consider *f*_3_(*x*) = *e* ^*x*^3^−*x*^ − cos⁡(*x* ^2^ − 1) + *x* ^3^ + 1, α = −1, *x*_0_ = −1.65, β_0_ = −1.

Methods with memory	|*x*_1_ − α|	|*x*_2_ − α|	|*x*_3_ − α|	coc
Our method (23)	6.0256(−3)	4.7294(−38)	3.8067(−459)	11.9952
Our method (24)	5.9797(−3)	4.4345(−38)	1.7580(−459)	11.9955
Our method (25)	6.0104(−3)	4.6300(−38)	2.9502(−459)	11.9953
Our method (26)	5.9959(−3)	4.5324(−38)	2.2850(−459)	11.9954
Our method (27)	5.3628(−3)	9.5497(−39)	1.7425(−467)	11.9929
Our method (28)	5.3357(−3)	9.1483(−39)	1.0410(−467)	11.9931
Our method (29)	5.3538(−3)	9.4151(−39)	1.4696(−467)	11.9929
Our method (30)	5.3448(−3)	9.2821(−39)	1.2391(−467)	11.9930
(31), β = −1	1.3000(−2)	5.5214(−33)	1.2114(−401)	12.1381
(32), β = −1	1.0937(−2)	3.5221(−34)	1.7174(−415)	12.1081
